# Leukocyte Tracking Database, a collection of immune cell tracks from intravital 2-photon microscopy videos

**DOI:** 10.1038/sdata.2018.129

**Published:** 2018-07-17

**Authors:** Diego Ulisse Pizzagalli, Yagmur Farsakoglu, Miguel Palomino-Segura, Elisa Palladino, Jordi Sintes, Francesco Marangoni, Thorsten R. Mempel, Wan Hon Koh, Thomas T. Murooka, Flavian Thelen, Jens V. Stein, Giuseppe Pozzi, Marcus Thelen, Rolf Krause, Santiago Fernandez Gonzalez

**Affiliations:** 1 Institute for Research in Biomedicine (IRB), Università della Svizzera italiana. Via Vincenzo Vela 6, 6500 Bellinzona, Switzerland; 2 Institute of Computational Science (ICS), Università della Svizzera italiana. Via Giuseppe Buffi 13, 6900 Lugano, Switzerland; 3 IMIM Hospital del Mar Medical Research Institute. Dr. Aiguader, 88, 08003 Barcelona, Spain; 4 Center for Immunology and Inflammatory Diseases, Massachusetts General Hospital. CNY 149-8 149 13th Street Charlestown, MA 02129, USA; 5 Department of Immunology, University of Manitoba. 471 Apotex Centre 750 McDermot Avenue, Winnipeg, MB R3E 0T5, Canada; 6 Theodor Kocher Institute (TKI), University of Bern. Freiestrasse 1, 3012 Bern, Switzerland; 7 Dipartimento di Elettronica, Informazione e Bioingegneria, Politecnico di Milano. P.za L da Vinci 32, I-20133 Milano, Italy

**Keywords:** Data mining, Image processing, Immunology, Systems biology, Machine learning

## Abstract

Recent advances in intravital video microscopy have allowed the visualization of leukocyte behavior *in vivo*, revealing unprecedented spatiotemporal dynamics of immune cell interaction. However, state-of-the-art software and methods for automatically measuring cell migration exhibit limitations in tracking the position of leukocytes over time. Challenges arise both from the complex migration patterns of these cells and from the experimental artifacts introduced during image acquisition. Additionally, the development of novel tracking tools is hampered by the lack of a sound ground truth for algorithm validation and benchmarking. Therefore, the objective of this work was to create a database, namely LTDB, with a significant number of manually tracked leukocytes. Broad experimental conditions, sites of imaging, types of immune cells and challenging case studies were included to foster the development of robust computer vision techniques for imaging-based immunological research. Lastly, LTDB represents a step towards the unravelling of biological mechanisms by video data mining in systems biology.

## Background & Summary

**Figure 1:**
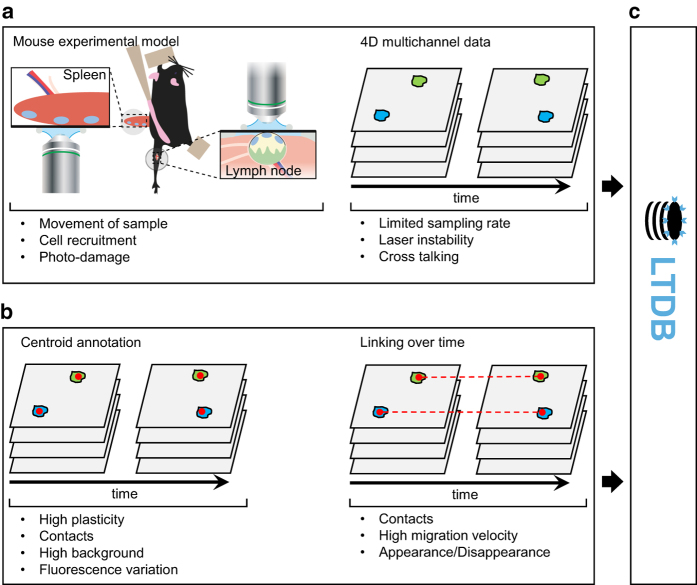
Data generation workflow. (**a**) *in vivo* imaging acquisition (left) Surgically exposed tissues from an anaesthetized and immobilized mouse are subjected to MP-IVM. (right) 4D data composed by z-stack of parallel image planes are acquired at different time points for multiple channels. (**b**) Cell detection and tracking (left) Cells are detected and the centroid position annotated the image series (red dots). Subsequently, (right) centroids are associated over time producing the cell tracks. For each phase of the imaging pipeline are reported specific problematics that affect cell tracking. **c**. Database. Both 4D imaging data and cell tracks are included in LTDB.

Multi-Photon Intravital Video Microscopy (MP-IVM), in combination with image-based systems biology^1^, represent a key methodology for studying the interplay of cells in organs and tissues of living animals^2^. Indeed, recent analyses of leukocyte migration in MP-IVM data, highlighted unprecedented cell-to-cell interaction patterns such as antigen capturing^3^ and presentation^4^, host-pathogen interaction^5,6^, tumor immune surveillance^7^ and cell activation^8^ amongst others. The advantage of MP-IVM with respect to other optical methods relies on the usage of multiple infrared photons. The low energy of the photons allows a deep and point-wise excitation of the sample which reduces light scattering and limits photo-damage. These properties make MP-IVM suitable to capture 4D data with remarkable resolution, depth and prolonged periods of observation^9^. The most common image acquisition and analysis pipeline of MP-IVM data is depicted in (Fig. 1). Initially, an animal having fluorescent cells, is anaesthetized, and prepared for imaging by immobilization and surgical exposition of the organ of interest (Fig. 1a left). Then, 4D data, composed of parallel image planes at different depths, are acquired for several time instants (Fig. 1a right). After acquisition, data are analysed by detecting cells (Fig. 1b left), tracking their position over time (Fig. 1b right) and finally quantifying cell migration^10^. The described pipeline was used to generate all the entries proposed in the current work (Fig. 1d).

**Figure 2:**
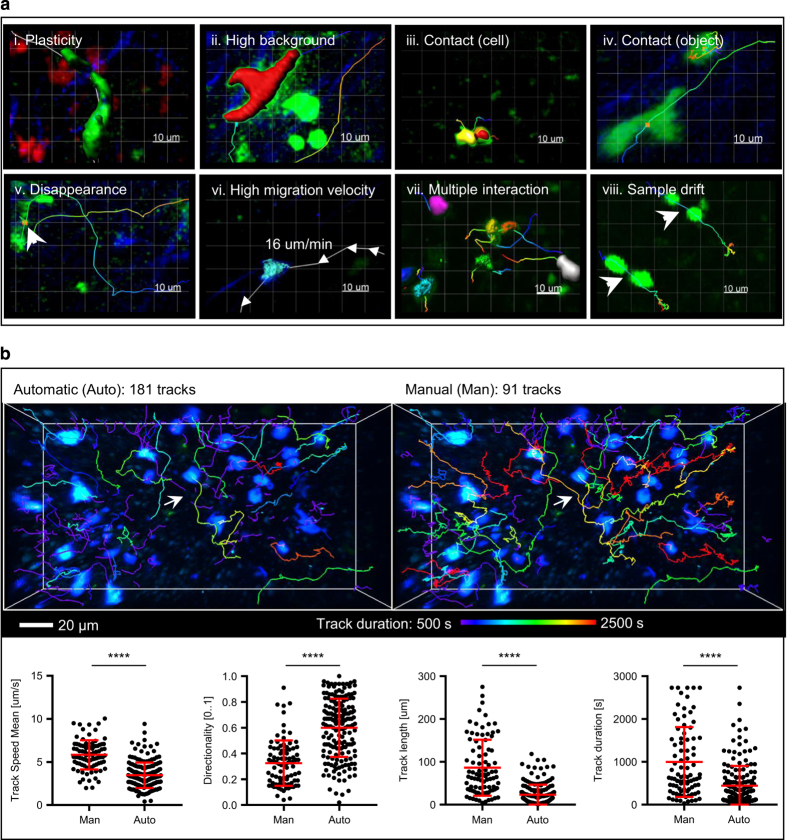
Tracking challenges. (**a**) Example case studies (i-viii) Representative snapshots of selected MP-IVM micrographs from problematic cases indicated in the upper part of the picture. (i) Surface reconstruction (SR) (green) of a T cell with uropodia. (ii) SR (red) of a T cell migrating with pseudopodia on a high background. (iii) SR (yellow, red) of two neutrophils forming a brief contact. (iv) Centers of mass (red dots) of a T cell forming a brief contact with a non motile object. (v) Estimated center of mass (red dot) of a T cell close to a boundary of the field of view. (vi) SR (blue) of a rapidly migrating Neutrophil. Arrows indicate cell displacement. (vii) SR (colored objects) of Neutrophils forming multiple contacts. (viii) Tracks (colored lines) of two B cells. (**b**) Effect of tracking errors Tracking errors limit research reproducibility, significantly (p≤0.0001) affecting the typical readouts from MP-IVM experiments. The figures (top-left and top-right) and the graphs (bottom) compare the manual tracks presented in *LTDB017_a* vs. the tracks generated automatically by Imaris. Automatic tracks were interrupted when the software could not detect or link cells, yielding to the creation of an increased number of shorter tracklets.

Despite the existence of specialized imaging software packages such as Imaris (Bitplane), Volocity (PerkinElmer) and FIJI^11^, the automatic analysis of immune cell migration^10^ in MP-IVM data is problematic. Challenges are introduced at each stage of the previously described pipeline and arise both from the complex biomechanical properties of leukocytes and from technical artifacts of *in vivo* imaging (Fig. 1,Table 1 and Fig. 2a). More specifically, high plasticity of cell shape, sustained speed and frequent contacts, set a limit on the capacity of detecting and tracking cells for long time periods^12^. Additionally, technical artifacts such as the variation and non-uniform diffraction of the light emitted by fluorescently- tagged cells or the physiological movement of the sample due to peristalsis, breathing or pulsing of blood vessels, further challenge the automatic analysis. Therefore, additional steps such as image pre-processing, tuning of software parameters and manual curation of tracks, are required to improve tracking results. As a consequence, usability of imaging software is reduced^13^, bias introduced and the reproducibility of the results is compromised. An example is provided in (Fig. 2b) where the Track Speed Mean, Directionality, Track length and Track duration were computed for the entry LTDB017a (Data Citation 1). These values exhibited highly significant differences (p≤0.0001) between automatically-generated vs. manually-generated tracks.

Providing the scientific community with datasets interpreted by experts is essential to foster the development of data science methods. To this end, international cell tracking challenges on public datasets^14,15^ allowed to highlight the properties amongst different algorithms. However, the provided datasets did not include leukocytes observed by intravital imaging. For this reason, it is necessary to develop an extended dataset of MP-IVM videos, where a significant number of leukocytes are tracked.Here we present a leukocyte tracking database, namely "LTDB", that includes MP-IVM videos of immune cells, together with their relative tracks which were manually annotated by experts. Each video contains one or more challenges for the automatic analysis (Table 2 (available online only)), and captured the behaviour of one or more cell populations (Table 3) in response to different stimuli (Table 4).

**Figure 3:**
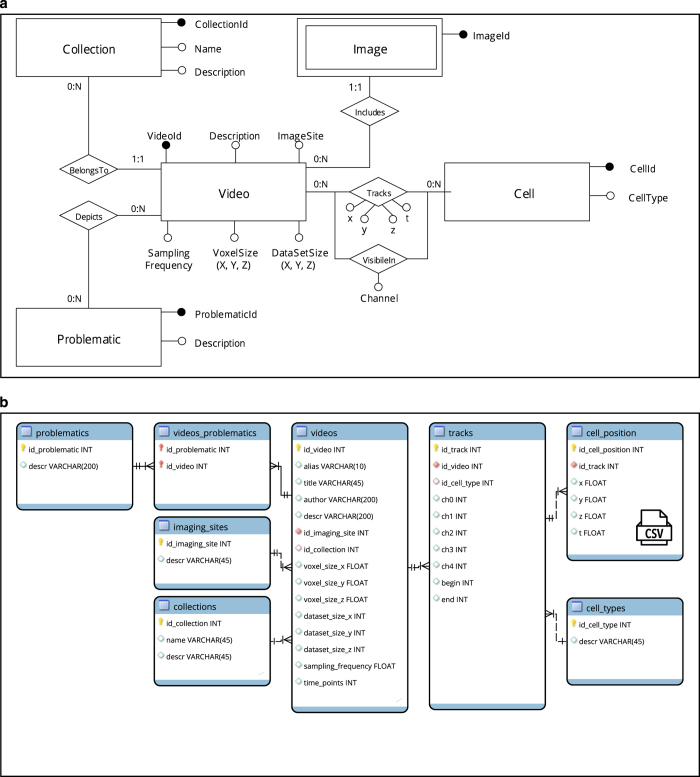
Data organization and formats. Videos, metadata and the position over time of tracked cells are organized as the conceptual Entity-Relationship model described in (**a**), corresponding to the logical database (optimized version) depicted in (**b**). A video belongs to a Collection, depicts one or more Problematic and includes an Image series. The Image entity is double-framed because it is a weak entity, which depends on the Video entity. A Cell has one type and one unique identifier. One Video tracks one (or more) Cell, every Cell being depicted by the Track association at a given timestamp (t) and in a spatial position (x, y, z) of that Video. The VisibleIn association further describes the channel of the video in which a cell is visible. The logical database is derived from the conceptual model and then optimized for read-access. The cells_positions table stores the instantaneous coordinates of each cell and is pre-exported in one or more CSV text files for each video. Imaging data are logically stored as TIFF image series with a specific filename.

All the videos and tracks are made available as individual files or as a spatio-temporal database (Fig. 3a) which was optimized for faster access to data and metadata (Fig. 3b).

**Figure 4:**
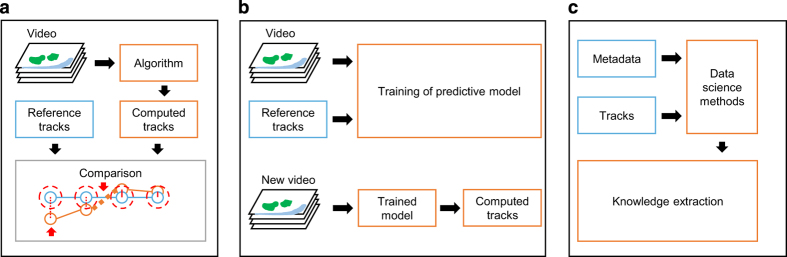
Typical usage scenarios. (**a**) Evaluation of a tracking algorithm. LTDB videos are provided as input to a tracking algorithm. Computed tracks can be compared with respect to the ground-truth tracks using a methodology of choice such as the complete graph comparison^15,16^. In the example red arrows indicate errors where a cell was detected not sufficiently close, and when a track was interrupted. (**b**) Machine learning dataset. LTDB videos and tracks can potentially be used in the context of supervised machine learning as training and validation dataset. The generated predictive model can be generalized and used to track new videos. (**c**) Resource for big data analysis. Properties of leukocyte migration in different experimental conditions can potentially be discovered by the application of pattern recognition on LTDB metadata and tracks.

The expected usage of LTDB is to serve as a ground truth for the validation of tracking algorithms (Fig. 4a). Differences with respect to the ground truth can be evaluated using, for instance, a metric that accounts for complete tracking graph comparison^16^.

LTDB further aims at being a training dataset for supervised machine learning methods. Indeed, in light of the recent application of deep learning for object detection and tracking in highly variable scenarios^17–19^, LTDB can provide the large number of images-tracks pairs required for the training of predictive models (Fig. 4b). In this case, broad imaging conditions may support the generalization capabilities of these methods.

Although LTDB was provided to primarily enhance tracking algorithms, the database embeds biomedical knowledge. To this end, data-mining and image-based systems biology methods can be applied to correlate images, tracks and metadata for investigating properties of the immune system in health and disease (Fig. 4c).

## Methods

### Imaging data generation

Experiments were performed by four research groups using three customized two-photon microscopy platforms (Table 5). Either the splenic or the lymph-node surgical models were used for acquisition (Fig. 1a). Videos were acquired from 26 unique experiments, to observe the interplay of neutrophils, B cells, T cells and natural killer cells in innate or adaptive immune responses (Table 4).

### Data pre-processing

No image processing was applied to the provided videos. RAW images were also used for manual tracking. Cropping of large 4D volumes in space and/or time was performed for the entries of the case study collection to focus on the area of interest.

### Manual tracking

Centroids of cells were manually annotated and linked over time, using the "Spots drawing" tool from Imaris (Bitplane). This process was performed by a group of three operators who tracked all the cells independently, redundantly and in three different locations without seeing the results produced by each other. In order to maximize track duration, cells were tracked also if partially visible. Tracks were interrupted only when cells completely disappeared. For specific studies, tracks of partially visible cells, migrating close to the boundaries of the field of view, can be excluded a posteriori by the user. Videos with ID (LTDB001 to LTDB020) have the maximum number of visible cells tracked. Videos in the Case Study collection (CS001 to CS018), instead, have only selected and challenging cells tracked.

### Consensus tracking ground truth generation

Multiple independent annotations and tracks were merged into the consensus ground truth provided along with the dataset using a majority-voting scheme. This process was performed manually by a fourth expert using the "Unify" functionality of Imaris. The Matlab script LTDBCheck.m was used to facilitate track matching, detecting common errors and highlighting conflictive situations. Two tracks were said likely to "match" (i.e. referring to the same cell) if their annotations were closer than 10*um* for at least *N* time instants. *N* was defined as the minimum between the track duration and 10. Conflictive situations were detected as tracks matching for only certain time instants but not for the entire track duration. These include a) tracks with an annotation in a far position by mistake, b) a longer track matching with one or multiple shorter tracks, c) two tracks matching for *N* instants but having different initial and/or final positions (i.e. track switches for closely interacting cells) amongst others. Tracks with a duration shorter than 4 time instants were also inspected manually. Due to the high plasticity of cells these criteria were used only to facilitate the work of the fourth expert who had to manually merge multiple tracks as follows: If at least two operators agreed on the direction of a cell, the track was included in the dataset (i.e. two matching tracks having the same duration and detected in the same frames). If two operators tracked a cell, but the track duration was different, the points annotated only by one operator were evaluated, confirmed or discarded by the fourth expert. When two operators could not agree on the direction of a cell, the following method was applied. If the fourth expert or the Matlab script identified an evident tracking error (i.e. cells not annotated by mistake, unrealistic jumps or broken tracks) the error was corrected and the tracks were merged. For real conflictive situations (i.e. track switching for closely interacting cells) the experts were asked to meet and discuss the most appropriate solution. If still the majority consensus could not be reached, and only in this case, tracks were interrupted. Finally, the position of cell centroids included in the ground truth was not averaged but selected as the centroid closer to the mean. Although this choice may produce less smooth tracks, it avoids to position a centroid outside non-convex cells.

These criteria together with the manual merging of tracks and re-evaluation of tracking conflicts, allowed to include the maximum number of tracks for the longest possible period of time.

### Animal models

The mouse strains included in this study are specified in Table 6 (available online only).

Prior to imaging, mice were anesthetized with a cocktail of Ketamine (100 mg/Kg) and Xylazine (10 mg/Kg) as previously described^4^. All animals were maintained in specific pathogen-free facilities at the Institute for Research in Biomedicine (Bellinzona, CH), Theodor Kocher Institute (Bern, CH) and Massachusetts General Hospital (Boston, MA). All the experiments were performed according with the rules and regulations of the local authorities and approved by the institutional animal committees: Swiss Federal Veterinary Office, Research Animal Care of the Massachusetts General Hospital, MGH Institutional Animal Care and Use Committee (IACUC).

### Code availability

To facilitate the usage of LTDB, the following Matlab code is provided, under the GPL v3 Open Source licence, at http://www.ltdb.info/downloads/ or via Git-Hub at https://github.com/IRB-LTDB/.

LTDBReadTracks.m Reads the tracks contained in a CSV file.

LTDBReadImages.m Reads the 4D images contained in the TIFF files.

LTDBExampleQuery.m Provides an example for querying the locally installed database.

XTLTDBImportTracks.m Imports tracks as spots in Imaris.

LTDB2C2C.m Exports LTDB tracks in the format used for the Cell Tracking Challenge described in^14,15^, mainly for evaluation with the methods proposed in^15^.

LTDBCheck.m Checks for common tracking errors (i.e. annotations deleted by mistakes, broken tracks and overlapping tracks).

EstimateDSMeasures.m Estimates the measures in (Table 2 (available online only)) regarding the dataset complexity.

LTDBCheck.m and EstimateDSMeasures.m make use of the following libraries: ImarisReader (https://github.com/PeterBeemiller/ImarisReader) to read Imaris files and bwdistsc^20^ to efficiently estimate the distance of each voxel from the closest centroid.

## Data Records

Data included in this work (Videos and Tracks) are available through *figshare* (Data Citation 1)

Images resulting from MP-IVM are contained in two zip archives with name TIFFS_LTDB001_LTDB020.zip for the videos with ID 001 to 020, and in TIFFS_CS001_CS018.zip for the videos in the case study collection with ID 001 to 018. In these archives, a folder for each video contains 4D images as TIFF files.

Tracks resulting from the consensus tracking ground truth generation, are contained in the archive GT_TRACKS.zip

A dump of the SQL database used to organize data and metadata is provided in LTDB.sql.

The following supplementary files are available through *figshare* (Data Citation 1). For a quicker preview, each is available in a H264 encoded MP4 file named <VideoID>.mp4.

A snapshot of all the videos is contained in the archive SNAPSHOTS.zip respectively named <VideoID>.png

Individual tracks produced by different operators are provided in the supplementary archive operator_individual_tracks.zip and named <VideoID>_<TrackID>_<OpID>.csv. In this case <OpID> is the ID of the operator (OP1, OP2 or OP3).

## Technical Validation

### Imaging data

Imaging data were captured from organs of living animals using either the splenic or the popliteal lymph node surgical models (Fig. 1a and Table 4) which are typical for MP-IVM investigations of the immune system^2^. Cells involved in both innate and adaptive responses were included in the dataset. Videos 12, 13, 14 (Data Citation 1) come from recently published MP-IVM studies^5,7,21^. To represent data generated by multiple laboratories in different experimental settings^22^, LTDB includes videos with different size, resolution, sampling rate and challenges for the automatic analysis (Table 2 (available online only)), acquired by three different microscopy platforms (Table 5). Moreover, cells were labelled with different fluorescent tags and detected by multiple channels (Table 3).

The following measures were computed to estimate the complexity of each video: signal to noise ratio (SNR), minimum distance between two cells (Dist) and number of cells per time instant. Since the proposed dataset is centroid-based rather than segmentation-based, SNR was estimated by adapting the definitions proposed in^15^ with the following heuristic. Let *c*_*i,t*_ be the centroid position of cell *i* at time *t*. For each voxel *v* in the current frame, the distance to the closest centroid was computed as $d_{v}=\min(\mathrm{||}v-c_{i,t}\mathrm{||})\forall i$. Then, considering a typical cell diameter of 10*um*, each voxel *v* was defined as foreground (FG-inside a cell) or background (BG-outside a cell) according with (Equation 1). This assumption allowed to sample a sufficient number of points in each video to estimate the aforementioned measures. (Table 2 (available online only)) summarizes the average values of each video while the additional script EstimateDSMeasures.m can be used to compute the values for each time instant.

(1)v∈FG⇒dv<4umv∈BG⇒dv>20um

(2)SNR=||avg(FG)−avg(BG)||std(BG)

### Tracks

The consensus tracking ground truth provided with LTDB includes 728 unique tracks composed of 44722 instantaneous annotations. On average, each track is composed by 61 annotations. This varying with the track duration. The total observation time included in LTDB amounts to the equivalent of 260 hours for a single cell.

Common tracking errors (i.e. cells not annotated by mistake, broken tracks or jumps in the z-axis) as well as conflicts produced by multiple operators were detected by executing the Matlab script LTDBCheck.m provided in the code availability section.

Individual operators produced 1850 tracks (113807 annotations) which were merged into the 728 tracks of the consensus tracking ground truth. The performances of each operator with respect to the consensus ground truth is reported in (Table 7). To this end, the TRA^15^ measure was computed. This measure includes a complete comparison of tracks represented as an acyclic oriented graph^16^. In order to estimate this measure, the ground truth and the individual tracks were converted in the format described in^15^ and evaluated using the TRAMeasure software provided along. However, that software and methodology matches a cell in the ground-truth with a cell in the track to be evaluated, when they overlap more than 50% in space. Being our dataset centroid-based a difference of 1 voxel would made the matching not possible. Hence, considering the typical cell diameter, we approximated a sphere around each of the centroids. The tolerance radius of the spheres was at maximum of 10*um* and was truncated in case of two centroids closer than 10*um*. The script LTDB2CTC.m was used to export the LTDB tracks in the acyclic oriented graph format described in^14,15^.

## Usage Notes

The expected use case scenario of LTDB is the evaluation of results produced by a cell tracking algorithm (Fig. 3a). Considering a generic cell tracking algorithm as an input-output system that reads an image sequence and outputs the tracks, LTDB can be used both as a source of images and as a ground truth for comparing the output.

To assess the overall performances of a cell tracking algorithm, we direct the user of LTDB towards the entries LTDB001 to LTDB020. To test the behaviour of an algorithm on specific cases instead, we recommend the user with the videos in the Case Study collection CS001 to CS018 that facilitates manual investigation and debugging having a reduced number of cells.

4D images are provided as uint16 TIFF files. File names were structured as <VideoID>_Txxx_Cxxx_Zxxx.tiff where <VideoID> is either(LTDB001 to LTDB020 or CS001 to CS018), the suffix xxx after T, C, Z indicates time instant, channel number and depth level respectively and spans from 000 to 999 maximum. Images with a lower bit depth were stored as uint16 without any scaling. If needed, for normalization the bit-depth of each video can be found in (Table 2 (available online only)).

Tracks are provided in the CSV Format described in (Table 8) and named <VideoID>_<TrackID>_GT.csv. For videos with only one cell population <TrackID> is "a", while for videos with two cell populations tracked it is either "a" or "b". These suffixes correspond to the suffixes used in (Table 2 (available online only)).

The synthetic example with ID SQUARE was added to the dataset. This provides a test-case for software having different coordinate systems. A parallelogram of 5×5×10*um* is positioned in the first frame close to the origin used for LTDB videos, corresponding to the bottom(x=0), left(y=0), deepest(z=0) corner of the 3D volume. This parallelogram migrates along the y-axis.

In order to evaluate tracking performances we provide a Matlab script LTDB2CTC.m to export LTDB tracks as the acyclic oriented graph representation^16^ used in the Cell Tracking Challenge described in^14,15^. This allows the usage of the accurate methodology and software provided by the aforementioned authors to compare computed tracks vs. ground truth.

For detecting cell populations visible in more than one channel (Table 2 (available online only) and Table 3) we encourage the usage of a co-localization method based on supervised machine learning such as Ilastik^23^ or Trainable Weka Segmentation^24^.

For discriminative machine learning models, it is worth noticing that all the cells of the videos LTDB001 to LTDB020 which are expected to be visible in the indicated channels were tracked. Other objects such as background, cell debris or additional cell populations were not tracked.

In the context of big-data analysis, (Fig. 3c) LTDB represents a resource to compare the biological properties of tracks (i.e. speed, directionality) amongst different experimental conditions. A review of the possible measures that could be computed from the tracks is provided in^10^.

The SQL database ltdb.sql can be installed optionally and for instance using the MySQL database management system. Queries to retrieve videos of interest (i.e. associated to a specific challenge, type of cell or site of imaging) can be addressed to the locally installed database. Additionally, a web interface was set up to facilitate search, preview and download of videos and it is accessible at http://www.ltdb.info/

## Additional information

**How to cite this article**: Pizzagalli, D. U. *et al*. Leukocyte Tracking Database, a collection of immune cell tracks from intravital 2-photon microscopy videos. *Sci. Data* 5:180129 doi: 10.1038/sdata.2018.129 (2018).

**Publisher’s note**: Springer Nature remains neutral with regard to jurisdictional claims in published maps and institutional affiliations.

## Supplementary Material



## Figures and Tables

**Table 1:** Biomechanical and technical problems.

Problem	Description	Effect on cell detection and tracking
Plasticity (Pla)	High variability in cell shape, such as elongation and formation of protrusions	Parts of the same cell not detected or associated to other cells
Contact (Con)	Close proximity of two cells with the same color	Cells merged in a single object. Track interrupted or switched
High background or low signal to noise ratio (BG)	Background or other objects (collagen fibers, auto-fluorescence, cell debris) appear in the same channel of cells with a similar brightness	Inaccurate cell detection, track interruption, tracking of third objects
Fluorescence variation (Flu)	The intensity of fluorescent cells changes during acquisition. Reasons include photo-bleaching and migration in different areas of the tissue	Inaccurate cell detection and track interruption
High migrating velocity (Vel)	Migration velocity greater than the cell size in a time step (absence of overlap)	Track interruption and aliasing if assumptions for interpolation rules for poorly visible cells are not correct. Deformation of cell shapes
Appearance and Disappearance (A/D)	Sudden or progressive appearance/disappearance of a cell, either close to the boundaries of the field of view or in proximity to a blood or lymphatic vessel	Track duration is less or equal than the length of the video. Tracking errors if interpolation rules for poorly visible cells are not correct
Movement of the sample (Mov)	Shifting, drifting or fluctations of the sample due to the movement of the animal or insufficient isolation from breathing, peristalsis and heartbeat	Non-rigid deformation of the tissue, discontinuities in tracks
Microscope instability (Ins)	Noise introduced either by oscillations in the laser power or in the sensitivity of the photo-detectors, resulting in bands or bright spots	Detection of larger or smaller objects. Appearance of the background, disappearance of cells
Large areas (Lar)	Non-uniform brightness	Frequent detection and tracking errors if parameters are not adjusted locally. Increased computational time
Channel specificity (Spe)	Emitted spectrum is captured in more than one channel	Mis-detection and increased contacts with the background
Density (Den)	High number of cells in close proximity	Track switching for tightly interacting cells and mis-detection
Description of the main problems for automatic cell detection and tracking. In brackets it is reported the abbreviation used to refer at each specific problem.		

**Table 2:** Dataset overview Overview of the size and complexity of each video-track entry of LTDB.

	**DATASET SIZE**				**VOXEL SIZE**			**D. TYPE**		**SNR**				**COMPLEXITY**		**CHALLENGES**										
**CS**	**W**	**H**	**D**	**T**	**xy**	**z**	**t**	**bit**	**C**	**CH0**	**CH1**	**CH2**	**CH3**	**DIST**	**AT**	**Pla**	**Con**	**BG**	**Flu**	**Vel**	**A/D**	**Mov**	**Ins**	**Lar**	**Spf**	**Den**
001_a	127	83	11	31	1.15	4	15	12	4	0.1	0.6	**7.7**	0.3		1	**x**				**x**						
002_a	116	87	11	120	1.15	4	15	12	4	0.2	1.0	**11.5**	0.2		1	**x**			x		x					
003_a	116	86	11	120	1.15	4	15	12	4	0.2	0.6	**9.6**	0.3		1	**x**	x	x			**x**					
004_a	279	197	18	20	0.50	2	60	14	3	**14.4**	0.1	**17.0**			1	**x**				**x**					**x**	
005_a	241	200	14	29	0.43	3	32	14	1	**##**				12±3	1		**x**									
006_a	124	134	11	120	1.15	4	15	12	4	0.1	0.8	**9.7**	0.2	50±19	2	**x**	**x**									
007_a	300	320	14	29	0.43	3	32	14	1	**##**				11±4	12		**x**	x								
008_a	124	112	10	119	0.80	2	25	14	1	**##**				36±9	2							**x**				
009_a	108	76	11	115	0.80	2	25	14	2	**##**	1.3			10±2	4		**x**									
010_a	148	279	18	26	0.50	2	60	14	3	**2.5**	0.5	**3.0**		14±6	3	**x**	**x**								**x**	
011_a	218	187	16	60	0.85	2	30	14	3	**6.6**	0.4	0.2		47±25	3	**x**		**x**			x					
012_a	105	69	14	61	0.47	4	20	12	1	**4.3**				13±16	2			x								
013_a	256	144	14	39	0.27	2	30	14	3	**##**	0.2	**##**			1	x									**x**	
014_a	142	115	13	242	0.80	3	15	8	3	**##**	1.7	**8.2**		50±12	3	x			**x**		**x**				**x**	
015_a	279	116	10	220	0.50	3	12	8	3	0.5	**12.6**	0.8			1	**x**		**x**	**x**							
016_a	279	116	10	220	0.50	3	12	8	3	**2.8**	0.4	**6.5**			1	**x**	**x**	x			x				x	
017_a	163	132	10	128	0.50	3	12	8	3	**8.1**	1.7	**9.2**		28±14	2	**x**	**x**								x	
018_a	143	135	14	60	0.56	3	30	14	4	**2.8**	0.1	0.2	0.1	10±2	11	x	x						**x**			
**LTDB**	**W**	**H**	**D**	**T**	**xy**	**z**	**t**	**bit**	**C**	**CH0**	**CH1**	**CH2**	**CH3**	**DIST**	**AT**	**Pla**	**Con**	**BG**	**Flu**	**Vel**	**A/D**	**Mov**	**Ins**	**Lar**	**Spf**	**Den**
001_a	556	556	18	60	0.50	2	60	14	3	**4.0**	0.3	**4.9**		15±7	12	x	x	x		**x**				x	**x**	
002_a	556	556	18	59	0.50	2	60	14	3	**3.5**	0.3	**4.7**		13±5	14	x	x	x		**x**				x	**x**	
003_a	391	352	20	71	0.50	2	13	14	3	**14.3**	0.2	**6.9**		20±14	6				x			**x**			**x**	
004_a	555	555	14	135	0.80	3	15	8	3	**10.7**	1.2	**11.3**		36±12	7		x	**x**	x			**x**		x	**x**	
004_b	555	555	14	135	0.80	3	15	8	3	6.6	**16.7**	5.7		29±36	1		x	**x**				**x**		x		
005_a	555	555	15	131	0.80	3	15	8	3	**8.6**	2.2	**11.3**		43±14	7		x	**x**	x					x	**x**	
005_b	555	555	15	131	0.80	3	15	8	3	6.6	**11.0**	5.2		83±27	2		x	**x**	x					x		
006_a	555	555	14	138	0.80	3	15	8	3	**4.9**	1.1	**10.9**		120±10	3	**x**			x						**x**	
007_a	555	555	15	93	0.80	3	15	8	3	**3.5**	1.1	**8.7**		34±6	8		x	**x**	**x**					**x**	**x**	
007_b	555	555	15	93	0.80	3	15	8	3	**21.9**	3.1	6.1		15±5	29		x	**x**	**x**					**x**	**x**	
008_a	524	518	14	39	0.27	2	30	14	3	**15.8**	0.1	**8.5**			1								**x**		**x**	
009_a	391	352	20	23	0.50	2	14	14	3	**10.0**	0.1	**5.8**		55±15	4				x				x		x	
010_a	555	555	10	120	0.80	2	30	14	2	2.9	**##**			16±2	23				x					x		
011_a	725	725	10	45	0.38	2	40	14	2	**40.1**	1.1			16±2	9				**x**							
012_a	512	512	11	89	1.15	4	15	12	4	0.7	**8.5**	0.1	0.0	80±6	4	**x**		x	x					x		
012_b	512	512	11	89	1.15	4	15	12	4	0.1	1.9	**11.5**	0.1	16±5	39	**x**	**x**	x	x					x		**x**
013_a	512	512	11	120	1.15	4	15	12	4	0.0	0.5	**15.4**	0.2	10±3	58	**x**	**x**	x	x					x		**x**
014_a	257	257	11	241	0.60	4	15	12	4	0.1	0.5	0.2	**3.2**	14±6	6	**x**	**x**	x	x							**x**
014_b	257	257	11	241	0.60	4	15	12	4	**4.8**	0.2	0.2	0.1	12±3	14	**x**	**x**	x	x							**x**
015_a	523	523	16	61	0.38	4	20	14	4	0.1	**##**	6.3	0.1	20±5	12	x	x	x	x	**x**		x		x		
016_a	379	355	10	220	0.50	3	12	8	3	**5.7**	0.9	**12.6**		11±4	15	**x**	**x**	x							x	**x**
017_a	404	214	14	183	0.50	3	15	8	3	**14.7**	1.0	**31.8**		9±2	33	**x**	**x**	x							x	**x**
017_b	404	214	14	183	0.50	3	15	8	3	0.9	**8.4**	0.4		12±3	18	**x**	**x**	x	**x**						x	**x**
018_a	555	555	22	36	0.80	3	42	14	2	0.2	**##**			104±9	5								**x**	x		
019_a	430	409	22	80	0.80	3	30	14	3	**4.2**	0.4	**11.3**		32±21	6				**x**					x	**x**	
020_a	351	354	11	60	0.80	3	30	14	4	1.0	**7.6**	1.6	0.1		1							**x**				
The columns in the group DATASET_SIZE report the digital size of the 4D data (W: width, H: height, D: Depth, T: number of time instants). The columns in group VOXEL_SIZE express the physical size of each voxel(xy: along the axes x and y [*um*], z: distance between two image planes [*um*], t: time interval between to time instants [*s*]). The columns in the group D.TYPE (Data type) report the bit depth (bit), and the number of channels included. The columns in the group SNR report the average (over time) of the Signal to Noise Radio (SNR) for each channel in the video. When a cell population in not visible in a specific channel these values tends to 0 (empty if the channel does not exist in the current video). The SNR values of the channels in which a cell population is expected to be visible are in bold. The columns in COMPLEXITY group report the average minimum distance between cells (DIST) and the average number of cell present in each time instant (AT). The columns in the CHALLENGES group report the main challenges (bold **x**) and the secondary challenges included (regular x). Pla: high plasticity, Con: contacts, BG: high background/low SNR/presence of cell debris, Flu: fluorescence variation, Vel: high migration velocity, A/D: appearing/disappearing objects, Mov: movement of the sample, Ins: instabilities of the laser, Lar: large areas, Spf: channel specificity, Den: high density.																										

**Table 3:** Channel specification Description of which cell population is expected to be visible in each channel of the provided videos.

**VideoID**	**CH0**	**CH1**	**CH2**	**CH3**
CS001	BG(FR)	Tc(CMTMR)	**a:Tc(HIV-GFP)**	Coll
CS002	BG(FR)	Tc(CMTMR)	**a:Tc(HIV-GFP)**	Coll
CS003	BG(FR)	Tc(CMTMR)	**a:Tc(HIV-GFP)**	Coll
CS004	**a:Ne(UBC-CFP)**, Dc(CD11c YFP)	Vaccine	**a:Ne(UBC-CFP)**, Coll	
CS005	**a:Ne(UBC-GFP)**			
CS006	BG(FR)	Tc(CMTMR)	**a:Tc(HIV-GFP)**	Coll
CS007	**a:Ne(UBC-GFP)**			
CS008	**a:Ne(UBC-GFP)**			
CS009	**a:Ne(UBC-GFP)**			
CS010	**a:Ne(UBC-CFP)**, Dc(CD11c YFP)	Vaccine	**a:Ne(UBC-CFP)**, Coll	
CS011	**a:Ne(UBC-GFP)**, BG	BG	Coll, BG	
CS012	**a:Tc(CFSE)**			
CS013	**a:Ne(UBC-CFP)**, Dc(CD11c YFP)	Vaccine	**a:Ne(UBC-CFP)**, Coll	
CS014	**a:Ne(UBC-GFP)**	Vaccine	**a:Ne(UBC-CFP)**, Coll	
CS015	**a:Ne(UBC-CFP)**, Dc(CD11c YFP), AF	Ne(CMTMR), AF	**a:Ne(UBC-CFP)**, Coll, AF	
CS016	**a:Ne(UBC-CFP)**, Dc(CD11c YFP), AF	Ne(CMTMR), AF	**a:Ne(UBC-CFP)**, Coll, AF	
CS017	**a:Ne(UBC-CFP)**, Dc(CD11c YFP), AF	Ne(CMTMR), AF	**a:Ne(UBC-CFP)**, Coll, AF	
CS018	**a:NK(NCR1 GFP)**, Mp(CD169 Pe)	Mp(Cd169 Pe)	Vaccine	Coll
LTDB001	**a:Ne(UBC-CFP)**, Dc(CD11c YFP)	Vaccine	**a:Ne(UBC-CFP)**, Coll	
LTDB002	**a:Ne(UBC-CFP)**, Dc(CD11c YFP)	Vaccine	**a:Ne(UBC-CFP)**, Coll	
LTDB003	**a:Ne(UBC-CFP)**, Dc(CD11c YFP)	Vaccine	**a:Ne(UBC-CFP)**, Coll	
LTDB004	Ne(UBC-GFP), **a:Ne(UBC-CFP)**, AF	**b:Ne(CMTMR)**, AF	**a:Ne(UBC-CFP)**, Coll, AF	
LTDB005	Ne(UBC-GFP), **a:Ne(UBC-CFP)**, AF	**b:Ne(CMTMR)**, AF	**a:Ne(UBC-CFP)**, Coll, AF	
LTDB006	**a:Ne(UBC-CFP)**, Dc(CD11c YFP)	Vaccine, BG	**a:Ne(UBC-CFP)**, Coll	
LTDB007	**b:Ne(UBC-GFP)**, **a:Ne(UBC-CFP)**, AF	Ne(CMTMR), AF	**a:Ne(UBC-CFP)**, Coll, AF	
LTDB008	**a:Ne(UBC-CFP)**, Dc(CD11c YFP)	Vaccine	**a:Ne(UBC-CFP)**, Coll	
LTDB009	**a:Ne(UBC-CFP)**, Dc(CD11c YFP)	Vaccine	**a:Ne(UBC-CFP)**, Coll	
LTDB010	Ne(UBC-GFP), BG	**a:Bc(CTV)**		
LTDB011	**a:Ne(UBC-GFP)**, BG	Bc(CD19 RFP)		
LTDB012	BG(FR)	**a:Tc(CMTMR)**	**b:Tc(HIV-GFP)**	Coll
LTDB013	BG(FR)	Tc(CMTMR)	**a:Tc(HIV-GFP)**	Coll
LTDB014	**b:Bc(Bodipy 650)**	Tc(H2B-RFP)	Tc(NFAT-GFP)	**a:Bc(Ag+ Hoechst33342)**
LTDB015	Tc(CMAC)	**a:Tc(CFSE)**	Tc(CMTMR)	HEV(Meca-633)
LTDB016	**a:Ne(UBC-CFP)**, Dc(CD11c YFP)	Ne(CMTMR), AF	**a:Ne(UBC-CFP)**, Coll, AF	
LTDB017	**a:Ne(UBC-CFP)**, Dc(CD11c YFP)	Ne(CMTMR), AF	**a:Ne(UBC-CFP)**, Coll, AF	
LTDB018	Dc(CD11c YFP)	**a:NK(CMTMR)**		
LTDB019	**a:NK(CTV)**, Dc(CD11c YFP)	Vaccine	**a:NK(CTV)**	
LTDB020	BG	**a:NK(CMTMR)**	BG	Coll
Bold indicates the cells that have been tracked. Brackets reports the type of staining used. The prefix a: or b: is used to indicate which is the corresponding tracking file (e.g. for video 04, b:N.(CMTMR) means that the tracks relative to the neutrophils labelled with CMTMR are in 04b.csv). Legend: Bc = B cells, Tc = T cells, Ne = neutrophils, Dc = dendritic cells, NKs= natural killer cells, Mp = macrophages, AF = auto-fluorescence, BG = background, Coll = collagen.				

**Table 4:** Experimental settings.

VideoID	Site of Imaging	Immune stimulus	Group	Ref.
CS001	popliteal lymph node	HIV-infected humanized T cell	T.M. / T.T.M.	^5,25^
CS002	popliteal lymph node	HIV-infected humanized T cell	T.M. / T.T.M.	^5,25^
CS003	popliteal lymph node	HIV-infected humanized T cell	T.M. / T.T.M.	^5,25^
CS004	popliteal lymph node	Influenza Vaccine	S.F.G.	
CS005	spleen	Vaccinia Virus	S.F.G.	
CS006	popliteal lymph node	HIV-infected humanized T cell	T.M. / T.T.M.	^5,25^
CS007	spleen	Vaccinia Virus	S.F.G.	
CS008	spleen	Ovalbumin	S.F.G.	
CS009	spleen	Ovalbumin	S.F.G.	
CS010	popliteal lymph node	Influenza Vaccine	S.F.G.	
CS011	popliteal lymph node	Influenza Vaccine	S.F.G.	
CS012	popliteal lymph node	Steady State	J.V.S.	
CS013	popliteal lymph node	Influenza Vaccine	S.F.G.	
CS014	popliteal lymph node	Influenza Vaccine	S.F.G.	
CS015	popliteal lymph node	Influenza Vaccine	S.F.G.	
CS016	popliteal lymph node	Influenza Vaccine	S.F.G.	
CS017	popliteal lymph node	Influenza Vaccine	S.F.G.	
CS018	popliteal lymph node	Influenza Vaccine	S.F.G.	
LTDB001	popliteal lymph node	Influenza Vaccine	S.F.G.	
LTDB002	popliteal lymph node	Influenza Vaccine	S.F.G.	
LTDB003	popliteal lymph node	Influenza Vaccine	S.F.G.	
LTDB004	popliteal lymph node	Influenza Vaccine	S.F.G.	
LTDB005	popliteal lymph node	Influenza Vaccine	S.F.G.	
LTDB006	popliteal lymph node	Influenza Vaccine	S.F.G.	
LTDB007	popliteal lymph node	Influenza Vaccine	S.F.G.	
LTDB008	popliteal lymph node	Influenza Vaccine	S.F.G.	
LTDB009	popliteal lymph node	Influenza Vaccine	S.F.G.	
LTDB010	spleen	Ovalbumin	S.F.G.	
LTDB011	spleen	Ovalbumin	S.F.G.	
LTDB012	popliteal lymph node	HIV-infected humanized T cell	T.M. / T.T.M.	^5,25^
LTDB013	popliteal lymph node	HIV-infected humanized T cell	T.M. / T.T.M.	^5,25^
LTDB014	popliteal lymph node	Ovalbumin	T.M.	^7^
LTDB015	popliteal lymph node	Steady State	J.V.S.	
LTDB016	popliteal lymph node	Influenza Vaccine	S.F.G.	
LTDB017	popliteal lymph node	Influenza Vaccine	S.F.G.	
LTDB018	popliteal lymph node	Influenza Vaccine	S.F.G.	
LTDB019	popliteal lymph node	Influenza Vaccine	S.F.G.	
LTDB020	popliteal lymph node	Influenza Vaccine	S.F.G.	
Experimental conditions for each video. Ref. indicates the references for videos which are part of published works. The acronyms under the Group column correspond to S.F.G.: Santiago Fernandez Gonzalez group, T.M. / T.T.M.: Thorsten Mempel and Thomas T. Murooka group, J.V.S.: Jens V. Stein group				

**Table 5:** Microscopy platforms.

Microscope	Equipment	Format	VideoID
TrimScope (LaVision BioTec GmbH)	Ti:Sapphire lasers (Chamaleon Ultra I, Chamaleon Ultra II, CoherentInc.).Optical parametric oscillator (Chamaleon Compact OPO, Coherent Inc.), with 1010-1340nm emission and 690-1080nm output wavelength.	14 bits, 8 bits	CS004, CS005,CS007-CS011,CS013-CS018LTDB001-LTDB011,LTDB016-LTDB020
Ultima IV multiphoton microscope (Bruker Systems)	DeepSee and MaiTai Ti:Sapphire lasers (Newport/Spectra-Physics) tuned between 850 and 990 nm.	12 bits	CS001-CS003,CS006;LTDB012-LTDB014
TrimScope (LaVision BioTec GmbH)	MaiTai Ti:Sapphire laser (Spectraphysics) tuned to 780 or 840 nm. Trimscope I based on BX50WI fluorescence microscope (LaVisionBiotec).	14 bits	CS012LTDB015
Technical specifications of the MP-IVM microscopy platforms used to generate each video.			

**Table 6:** Mouse strains Specification of mouse strains used as host and as source of cells for each video.

VideoID	Host	Host provider	Host reference	Cells	Cell source	Cell source reference	Comment
CS001	BLT NOD.scid humanized mice	MGH Humanized mouse core	https://www.ncbi.nlm.nih.gov/pubmed/19420076	CD4+ T cells	BLT NOD.scid humanized mice	https://www.ncbi.nlm.nih.gov/pubmed/19420076	central memory-like CD4+ T cells were isolated and expanded from syngeneic BLT humanized mice, infected with HIV-GFP or labeled with Celltracker Orange (CMTMR) and injected to the host
CS002	BLT NOD.scid humanized mice	MGH Humanized mouse core	https://www.ncbi.nlm.nih.gov/pubmed/19420076	CD4+ T cells	BLT NOD.scid humanized mice	https://www.ncbi.nlm.nih.gov/pubmed/19420076	central memory-like CD4+ T cells were isolated and expanded from syngeneic BLT humanized mice, infected with HIV-GFP or labeled with Celltracker Orange (CMTMR) and injected to the host
CS003	BLT NOD.scid humanized mice	MGH Humanized mouse core	https://www.ncbi.nlm.nih.gov/pubmed/19420076	CD4+ T cells	BLT NOD.scid humanized mice	https://www.ncbi.nlm.nih.gov/pubmed/19420076	central memory-like CD4+ T cells were isolated and expanded from syngeneic BLT humanized mice, infected with HIV-GFP or labeled with Celltracker Orange (CMTMR) and injected to the host
CS004	CD11c-EYFP	Jackson	https://www.jax.org/strain/08829	Neutrophils	UBC-CFP mice	https://www.jax.org/strain/004218	Neutrophils expressing CFP were isolated from CK6/ECFP mice and injeted to the host animal
CS005	C57BL/6Jrj	Janvier laboratories	https://www.janvier-labs.com/rodent-research-models-services/research-models/per-species/inbred-mice/product/c57bl6jrj.html	Neutrophils	UBC-GFP mice	https://www.jax.org/strain/004353	Neutrophils expressing GFP were isolated from UBC-GFP mice and injected to the host animal
CS006	BLT NOD.scid humanized mice	MGH Humanized mouse core	https://www.ncbi.nlm.nih.gov/pubmed/19420076	CD4+ T cells	BLT NOD.scid humanized mice	https://www.ncbi.nlm.nih.gov/pubmed/19420076	central memory-like CD4+ T cells were isolated and expanded from syngeneic BLT humanized mice, infected with HIV-GFP or labeled with Celltracker Orange (CMTMR) and injected to the host
CS007	C57BL/6Jrj	Janvier laboratories	https://www.janvier-labs.com/rodent-research-models-services/research-models/per-species/inbred-mice/product/c57bl6jrj.html	Neutrophils	UBC-GFP mice	https://www.jax.org/strain/004353	Neutrophils expressing GFP were isolated from UBC-GFP mice and injected to the host animal
CS008	LysMcre GFP	Jackson	https://www.jax.org/strain/004781	Neutrophils	host		
CS009	CD19-RFP	Thelen Lab, IRB Bellinzona	https://www.ncbi.nlm.nih.gov/pubmed/17171761	Neutrophils	LysMcre GFP mice	Gonzalez lab, IRB Bellinzona	Neutrophils expressing GFP were isolated from UBC-GFP mice and injected to the host animal
CS010	CD11c-EYFP	Jackson	https://www.jax.org/strain/08829	Neutrophils	UBC-CFP mice	https://www.jax.org/strain/004218	Neutrophils expressing CFP were isolated from CK6/ECFP mice and injeted to the host animal
CS011	LysMcre GFP	Jackson	https://www.jax.org/strain/004781	Neutrophils	host		
CS012	C57BL/6J	Charles River	https://www.criver.com/products-services/find-model/jax-c57bl6j-mice?region=3616	T Cells	Ndr DKO mice	TKI, Bern	Ndr DKO mice were generated by crossing Ndr1−/−Ndr2f/f mice with mice expressing cre recombinase driven by the lck proximal promoter [Jackson Laboratory, B6.Cg-Tg (Lck-cre; 548Jxm/J stock #003802)]. All of the mice used for experiments were backcrossed to C57BL/6J mice for at least six generations. The C57BL/6J mice used for adoptive trans- fer experiments were purchased from Charles River.
CS013	CD11c-EYFP	Jackson	https://www.jax.org/strain/08829	Neutrophils	UBC-CFP mice	https://www.jax.org/strain/004218	Neutrophils expressing CFP were isolated from CK6/ECFP mice and injeted to the host animal
CS014	C57BL/6Jrj	Janvier laboratories	https://www.janvier-labs.com/rodent-research-models-services/research-models/per-species/inbred-mice/product/c57bl6jrj.html	Neutrophils	UBC-CFP mice, UBG-GFP mice	https://www.jax.org/strain/004218, https://www.jax.org/strain/004353	Neutrophils expressing CFP were isolated from CK6/ECFP mice and neutrophils expressing GFP were isolated from UBC-GFP mice. Both were injeted to the host animal. Additionally, the host animal was injected with CD169 PE
CS015	CD11c-EYFP	Jackson	https://www.jax.org/strain/08829	Neutrophils	UBC-CFP mice, P2rx7-/- mice	https://www.jax.org/strain/004218, https://www.jax.org/strain/005576	Cells were isolated from a UBC-CFP animal and from a P2rx7-/- animal labeled with CMTMR, then injected to the host animal
CS016	CD11c-EYFP	Jackson	https://www.jax.org/strain/08829	Neutrophils	UBC-CFP mice, P2rx7-/- mice	https://www.jax.org/strain/004218, https://www.jax.org/strain/005576	Cells were isolated from a UBC-CFP animal and from a P2rx7-/- animal labeled with CMTMR, then injected to the host animal
CS017	CD11c-EYFP	Jackson	https://www.jax.org/strain/08829	Neutrophils	UBC-CFP mice, P2rx7-/- mice	https://www.jax.org/strain/004218, https://www.jax.org/strain/005576	Cells were isolated from a UBC-CFP animal and from a P2rx7-/- animal labeled with CMTMR, then injected to the host animal
CS018	Ncr1 GFP	Jackson	https://www.jax.org/strain/022739	Natural killer cells	host		
LTDB001	CD11c-EYFP	Jackson	https://www.jax.org/strain/08829	Neutrophils	UBC-CFP mice	https://www.jax.org/strain/004218	Neutrophils expressing CFP were isolated from CK6/ECFP mice and transfected to the host animal
LTDB002	CD11c-EYFP	Jackson	https://www.jax.org/strain/08829	Neutrophils	UBC-CFP mice	https://www.jax.org/strain/004218	Neutrophils expressing CFP were isolated from CK6/ECFP mice and transfected to the host animal
LTDB003	CD11c-EYFP	Jackson	https://www.jax.org/strain/08829	Neutrophils	UBC-CFP mice	https://www.jax.org/strain/004218	Neutrophils expressing CFP were isolated from CK6/ECFP mice and transfected to the host animal
LTDB004	C57BL/6Jrj	Janvier laboratories	https://www.janvier-labs.com/rodent-research-models-services/research-models/per-species/inbred-mice/product/c57bl6jrj.html	Neutrophils	UBC-CFP mice, UBC-GFP mice, C57BL/6Jrj mice	https://www.jax.org/strain/004218, https://www.jax.org/strain/004353, https://www.janvier-labs.com/rodent-research-models-services/research-models/per-species/inbred-mice/product/c57bl6jrj.html	Neutrophils expressing CFP were isolated from CK6/ECFP mice, Neutrophils expressing GFP were isolated from UBC-GFP mice. Neuttrophils were isolated from B6 animals and labeled with CMTMR. The three different cells were injeted to the host animal.
LTDB005	C57BL/6Jrj	Janvier laboratories	https://www.janvier-labs.com/rodent-research-models-services/research-models/per-species/inbred-mice/product/c57bl6jrj.html	Neutrophils	UBC-CFP mice, UBC-GFP mice, C57BL/6Jrj mice	https://www.jax.org/strain/004218, https://www.jax.org/strain/004353, https://www.janvier-labs.com/rodent-research-models-services/research-models/per-species/inbred-mice/product/c57bl6jrj.html	Neutrophils expressing CFP were isolated from CK6/ECFP mice, Neutrophils expressing GFP were isolated from UBC-GFP mice. Neuttrophils were isolated from B6 animals and labeled with CMTMR. The three different cells were injeted to the host animal.
LTDB006	C57BL/6Jrj	Janvier laboratories	https://www.janvier-labs.com/rodent-research-models-services/research-models/per-species/inbred-mice/product/c57bl6jrj.html	Neutrophils	UBC-CFP mice, UBC-GFP mice, C57BL/6Jrj mice	https://www.jax.org/strain/004218, https://www.jax.org/strain/004353, https://www.janvier-labs.com/rodent-research-models-services/research-models/per-species/inbred-mice/product/c57bl6jrj.html	Neutrophils expressing CFP were isolated from CK6/ECFP mice, Neutrophils expressing GFP were isolated from UBC-GFP mice. Neuttrophils were isolated from B6 animals and labeled with CMTMR. The three different cells were injeted to the host animal.
LTDB007	C57BL/6Jrj	Janvier laboratories	https://www.janvier-labs.com/rodent-research-models-services/research-models/per-species/inbred-mice/product/c57bl6jrj.html	Neutrophils	UBC-CFP mice, UBG-GFP mice	https://www.jax.org/strain/004218, https://www.jax.org/strain/004353	Neutrophils expressing CFP were isolated from CK6/ECFP mice and neutrophils expressing GFP were isolated from UBC-GFP mice. Both were injeted to the host animal. Additionally, the host animal was injected with CD169 PE
LTDB008	CD11c-EYFP	Jackson	https://www.jax.org/strain/08829	Neutrophils	UBC-CFP mice	https://www.jax.org/strain/004218	Neutrophils expressing CFP were isolated from CK6/ECFP mice and transfected to the host animal
LTDB009	CD11c-EYFP	Jackson	https://www.jax.org/strain/08829	Neutrophils	UBC-CFP mice	https://www.jax.org/strain/004218	Neutrophils expressing CFP were isolated from CK6/ECFP mice and transfected to the host animal
LTDB010	LysMcre GFP	Jackson	https://www.jax.org/strain/004781	B Cells	LysMcre-GFP mice	Gonzalez lab, IRB Bellinzona	B Cells were isolated from non-GFP littermates (coming from the LysM-Cre-GFP crossings) and labelled with Celltracker violet
LTDB011	CD19-RFP	Thelen Lab, IRB Bellinzona	https://www.ncbi.nlm.nih.gov/pubmed/17171761	Neutrophils	LysMcre GFP mice	Gonzalez lab, IRB Bellinzona	Neutrophils expressing GFP were isolated from LysMcre-GFP mice and injected to the host animal
LTDB012	BLT NOD.scid humanized mice	MGH Humanized mouse core	https://www.ncbi.nlm.nih.gov/pubmed/19420076	CD4+ T cells	BLT NOD.scid humanized mice	MGH Humanized mouse core	central memory-like CD4+ T cells were isolated and expanded from syngeneic BLT humanized mice, labelled with Celltracker green and injected to the host
LTDB013	BLT NOD.scid humanized mice	MGH Humanized mouse core	https://www.ncbi.nlm.nih.gov/pubmed/19420076	CD4+ T cells	BLT NOD.scid humanized mice	MGH Humanized mouse core	central memory-like CD4+ T cells were isolated and expanded from syngeneic BLT humanized mice, infected with HIV-GFP or labeled with Celltracker Orange (CMTMR) and injected to the host
LTDB014	BALB/c	Jackson	https://www.jax.org/strain/017580	CD8+ T cells, Ag+ B cells, B Cells	CL4 mice, BALB/c mice	MGH, Jackson	Balb/c (Jackson) to which HA-specific CD8 (purified from CL4 mice, bred in house at Massachusetts General Hospital) transduced with NFAT-GFP and H2B-RFP were transferred 2 days before imaging. Upon imaging, two populations of differentially labeled B cells (purified from Balb/c mice, Jackson) were also transferred.
LTDB015	C57BL/6J	Charles River	https://www.criver.com/products-services/find-model/jax-c57bl6j-mice?region=3616	T Cells	Ndr DKO mice	TKI Bern	Ndr DKOmicewere generated by crossing Ndr1−/−Ndr2f/f mice with mice expressing cre recombinase driven by the lck proximal promoter [Jackson Laboratory, B6.Cg-Tg (Lck-cre; 548Jxm/J stock #003802)]. All of the mice used for experiments were backcrossed to C57BL/6J mice for at least six generations. The C57BL/6J mice used for adoptive trans- fer experiments were purchased from Charles River.
LTDB016	CD11c-EYFP	Jackson	https://www.jax.org/strain/08829	Neutrophils	UBC-CFP mice, P2rx7-/- mice	https://www.jax.org/strain/004218, https://www.jax.org/strain/005576	Cells were isolated from a UBC-CFP animal and from a P2rx7-/- animal labeled with CMTMR, then injected to the host animal
LTDB017	CD11c-EYFP	Jackson	https://www.jax.org/strain/08829	Neutrophils	UBC-CFP mice, P2rx7-/- mice	https://www.jax.org/strain/004218, https://www.jax.org/strain/005576	Cells were isolated from a UBC-CFP animal and from a P2rx7-/- animal labeled with CMTMR, then injected to the host animal
LTDB018	CD11c-EYFP	Jackson	https://www.jax.org/strain/08829	Natural killer cells	C57BL/6Jrj	Janvier laboratories	Natural killer cells were isolated from a B6 animal, labelled with CMTMR and injected to the host animal
LTDB019	CD11c-EYFP	Jackson	https://www.jax.org/strain/08829	Natural killer cells	C57BL/6Jrj	Janvier laboratories	Natural killer cells were isolated from a B6 animal, labelled with Celltracker violet and injected to the host animal
LTDB020	IFNg eYFP	Jackson	https://www.jax.org/strain/017580	Natural killer cells	C57BL/6Jrj	Janvier laboratories	Natural killer cells were isolated from a B6 animal, labelled with CMTMR and injected to the host animal

**Table 7:** Comparison of tracking operators Differences between the tracks produced by individual operators and the consensus ground truth, for all the videos in LTDB.

	TRA			Track duration				Number of tracks			
**CS**	**OP1**	**OP2**	**OP3**	**GT**	**OP1**	**OP2**	**OP3**	**GT**	**OP1**	**OP2**	**OP3**
**001_a**	1.00	0.87	0.86	30	30	30	30	1	1	1	1
**002_a**	0.90	0.98	1.00	119	119	79	119	1	1	2	1
**003_a**	0.99	0.99	1.00	118	117	118	118	1	1	1	1
**004_a**	1.00	1.00	1.00	18	18	18	18	1	1	1	1
**005_a**	0.99	1.00	1.00	22	22	22	22	2	2	2	2
**006_a**	1.00	0.96	0.95	119	119	118	119	2	2	2	2
**007_a**	0.61	1.00	0.69	26	27	26	28	13	7	13	8
**008_a**	1.00	0.99	0.99	111	111	90	91	2	2	3	3
**009_a**	1.00	1.00	1.00	114	114	114	114	4	4	4	4
**010_a**	1.00	0.99	0.99	25	25	22	22	3	3	4	4
**011_a**	0.84	1.00	0.75	37	44	37	35	6	4	6	5
**012_a**	0.85	1.00	0.93	15	16	14	16	9	7	10	8
**013_a**	1.00	1.00	0.98	38	38	38	38	1	1	1	1
**014_a**	0.38	0.95	0.96	185	179	88	203	4	1	9	5
**015_a**	1.00	0.97	0.98	184	184	179	180	1	1	1	1
**016_a**	0.97	0.97	1.00	155	153	157	155	1	1	1	1
**017_a**	1.00	1.00	1.00	116	116	116	116	2	2	2	2
**018_a**	1.00	0.59	0.48	42	40	52	42	15	16	7	7
**LTDB**	**OP1**	**OP2**	**OP3**	**GT**	**OP1**	**OP2**	**OP3**	**GT**	**OP1**	**OP2**	**OP3**
**001_a**	0.91	0.92	0.78	32	29	38	30	22	22	17	19
**002_a**	0.91	0.86	0.92	33	30	35	36	26	28	21	24
**003_a**	0.96	0.69	0.99	46	49	53	38	9	8	5	11
**004_a**	0.78	0.78	0.78	92	87	93	88	10	10	8	9
**004_b**	0.91	1.00	0.83	82	75	82	68	2	2	2	2
**005_a**	0.39	0.65	0.96	77	130	92	83	12	2	6	11
**005_b**	0.88	1.00	0.76	130	130	130	130	2	2	2	2
**006_a**	0.50	0.50	0.99	137	137	137	137	3	1	1	3
**007_a**	0.78	0.80	0.65	60	56	58	71	12	11	11	8
**007_b**	0.82	0.95	0.86	82	78	76	82	32	28	33	30
**008_a**	1.00	1.00	1.00	38	38	38	38	1	1	1	1
**009_a**	0.97	1.00	0.84	21	20	21	22	4	4	4	3
**010_a**	0.79	0.96	0.90	111	119	106	77	26	19	26	35
**011_a**	0.50	0.84	1.00	36	44	33	37	11	4	10	11
**012_a**	0.65	0.84	0.90	41	49	36	40	83	48	89	83
**012_b**	0.94	0.99	0.93	81	78	56	77	4	4	7	5
**013_a**	0.71	0.94	0.76	84	83	77	87	82	62	85	65
**014_a**	0.69	0.92	0.99	82	119	69	84	20	9	22	20
**014_b**	0.61	0.62	0.98	70	56	101	73	49	39	22	50
**015_a**	0.88	0.95	0.99	25	30	30	24	30	22	24	32
**016_a**	0.52	0.93	0.93	81	135	79	73	43	13	42	46
**017_a**	0.83	0.86	0.91	68	77	67	76	89	67	80	78
**017_b**	0.41	0.75	0.79	49	56	50	56	68	31	56	50
**018_a**	1.00	0.49	0.97	35	35	31	27	5	5	6	9
**019_a**	0.99	0.81	0.59	44	38	51	44	12	14	10	7
**020_a**	0.98	1.00	0.93	31	30	31	29	2	2	2	2
**average**	**0.84**	**0.89**	**0.90**	**71**	**74**	**68**	**70**	**17**	**12**	**15**	**15**
Values of the TRA measure [15] close to 1 means the accurate matching of the operators tracks with respect to the consensus ground truth while lower values indicates tracking differences.											

**Table 8:** Structure of the CSV track file.

Row 1	VideoID [string]	dx [um]	dy [um]	dz [um]	dt [s]
	*video identifier*	*voxel size (x)*	*voxel size (y)*	*voxel size (z)*	*time interval*
**Row 2**	**ch0 [bool]**	**ch1 [bool]**	**ch2 [bool]**	**ch3 [bool]**	**ch4 [bool]**
	*visible in channel 0*	*visible in channel 1*	*visible in channel 2*	*visible in channel 3*	*visible in channel 4*
**Rows 3 to end**	**TrackID [string]**	**x [um]**	**y [um]**	**z [um]**	**t [INT]**
	*unique track identifier*	*position (x)*	*position (y)*	*position (z)*	*time instant*
The position of the centroids of all the cells tracked in a video was saved in a ASCII CSV file. Columns are delimited by the semicolon separator and rows are terminated by CR LF. The first row reports the identifier of the video, including the eventual suffix "a" or "b". The second to the fifth columns report the voxel size (dx,dy,dz) and the time interval (dt). The second row specifies in which channel (Ch) cells appear in the video. From the third to the last row, the coordinates of cells are saved. The first column represents the unique identifier of a track, not varying for the entire track duration. The second to fifth columns (x, y, z, t) represent the position of the cell with respect to the top-up-left most corner of the z-stack at a specific time point. Coordinates are expressed in *μm* while the time point is an integer number.					
